# Tiny spies: mosquito antennae are sensitive sensors for eavesdropping on frog calls

**DOI:** 10.1242/jeb.245359

**Published:** 2023-12-11

**Authors:** Hoover Pantoja-Sánchez, Brian C. Leavell, Bianca Rendon, W. A. Priyanka P. de-Silva, Richa Singh, Jian Zhou, Gil Menda, Ronald R. Hoy, Ronald N. Miles, Neil D. Sanscrainte, Ximena E. Bernal

**Affiliations:** ^1^ Department of Biological Sciences, Purdue University, West Lafayette, IN 47907, USA; ^2^ Department of Environmental Toxicology, Texas Tech University, Lubbock, TX 41163, USA; ^3^ Department of Zoology, Faculty of Science, University of Peradeniya, 20400 Peradeniya, Sri Lanka; ^4^ Department of Mechanical Engineering, Binghamton University, Binghamton, NY 13902, USA; ^5^ Department of Neurobiology and Behavior, Cornell University, Ithaca, NY 14853, USA; ^6^ USDA Agricultural Research Service, Centre for Medical, Agricultural and Veterinary Entomology, Gainesville, FL 32608, USA; ^7^ Smithsonian Tropical Research Institute, Apartado 0843-03092, Balboa, Republic of Panamá

**Keywords:** Antenna, Bioacoustics, Communication networks, *Uranotaenia lowii*, Hearing, Midge

## Abstract

Most mosquito and midge species use hearing during acoustic mating behaviors. For frog-biting species, however, hearing plays an important role beyond mating as females rely on anuran calls to obtain blood meals. Despite the extensive work examining hearing in mosquito species that use sound in mating contexts, our understanding of how mosquitoes hear frog calls is limited. Here, we directly investigated the mechanisms underlying detection of frog calls by a mosquito species specialized on eavesdropping on anuran mating signals: *Uranotaenia lowii*. Behavioral, biomechanical and neurophysiological analyses revealed that the antenna of this frog-biting species can detect frog calls by relying on neural and mechanical responses comparable to those of non-frog-biting species. Our findings show that in *Ur. lowii*, contrary to most species, males do not use sound for mating, but females use hearing to locate their anuran host. We also show that the response of the antennae of this frog-biting species resembles that of the antenna of species that use hearing for mating. Finally, we discuss our data considering how mosquitoes may have evolved the ability to tap into the communication system of frogs.

## Teaser

**Highlighted Article:** The unique acoustic behavior of the frog-biting mosquito *Uranotaenia lowii*: intriguing insights into how these tiny spies eavesdrop on frog communication.

## INTRODUCTION

The mosquito antenna is recognized as one of the most complex ears found in insects (Robert and Hoy, 2009). Distinctive sensory characteristics support this affirmation. For instance, the number of cells in the sensory organ of the antenna is comparable to the number of hair cells in the human cochlea, but they are placed in a volume ≈100,000 times smaller. Such a unique trait has seized the attention of sensory biologists and engineers promoting research on mosquito bioacoustics. Most studies, however, have focused on the role of hearing for mating purposes, a context in which interactions occur at a close range (<50 cm) (Andrés et al., 2020). However, the use of hearing in mosquitoes is not limited to short distances. Some mosquito species rely on hearing to detect distant host cues (Bernal et al., 2006; Bernal and de-Silva, 2015; Borkent and Belton, 2006). But it is currently unclear how they are capable of sensing acoustic stimuli in this scenario. Although recent findings revealed the ability of antennal hearing systems to detect sound in the far field (Menda et al., 2019), the ecological relevance of such findings is unclear as antennal hearing has been only studied in species for which hearing seems restricted to interactions at short distances.

**Fig. 1.**
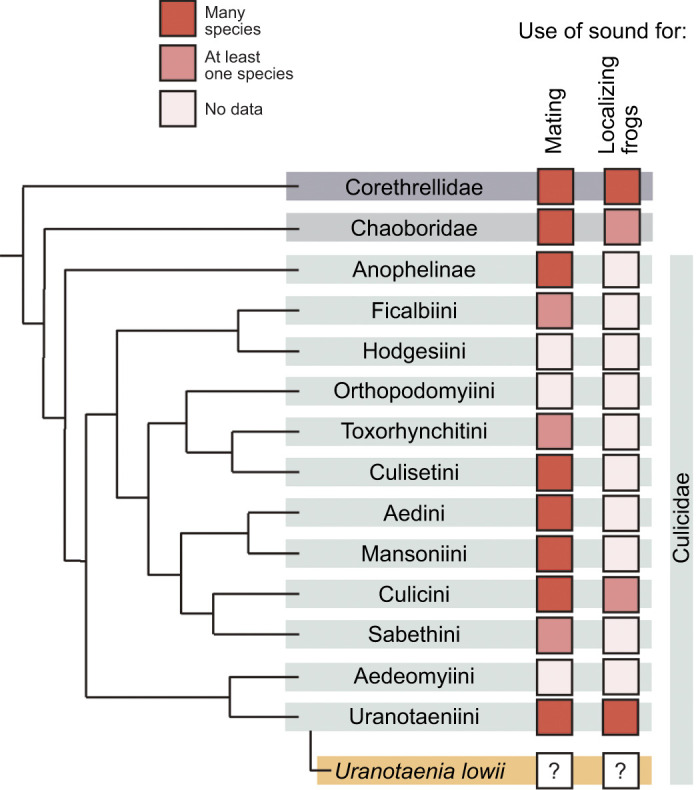
**Evolution of the use of sound across different contexts in mosquitoes and their relatives.** Use of acoustic mating signals and cues to localize frogs by mosquito (Culicidae) species and related linages: frog-biting midges (Corethrellidae) and phantom midges (Chaoboridae). Phylogeny adapted from Reeves et al. (2018) originally adapted from Harbach and Kitching (1998). References used in this figure are listed in Table S1.

The auditory system of mosquitoes provides an ideal opportunity to examine how antennal hearing allows detection of acoustic cues across different distances. Different species of mosquitoes depend on hearing in different biological contexts, such as mating and foraging, that require interactions across different spatial scales. The use of acoustic mating signals is a behavior widespread in mosquitoes (Culicidae) and their close relatives, phantom midges (Chaoboridae) and frog-biting midges (Corethrellidae). In most species, males and females form aerial aggregations and interact through the faint flight tones produced by the wingbeat of their mates (Downes, 1969). Indeed, acoustic mating signals are widespread among species in these families (Blackwell et al., 1992; Downes, 1969; Downes, 2009; Fedorova and Zhantiev, 2009; Fyodorova and Azovsky, 2003), and relying on sound for reproductive purposes is considered an ancestral trait in their infraorder (Culicomorpha) (de-Silva et al., 2015; Harbach and Kitching, 1998). Therefore, hearing plays a major role in reproduction in this clade. Although the use of sound in other contexts has received limited attention, a systematic review revealed that exploitation of frog calls occurs in at least 191 fly species across 23 genera (L. L. F. Campos, S. Neckel-Oliveira and X. E. B., unpublished data). Hearing, in this foraging context, is critical for females as it is necessary to support egg production. Eavesdropping on frog calls for foraging has evolved independently several times among mosquitoes and midges (Fig. 1), occurring in frog-biting midges (Corethrellidae), at least one species of phantom midge (Chaoboridae) and two tribes of mosquitoes (Culicini and Uranotaeniini) (Bartlett-Healy et al., 2008; Bernal and de-Silva, 2015; Borkent and Belton, 2006; Lopez-Méndez et al., 2015; Toma et al., 2005).

Many mosquitoes use their antennae to hear their mates and engage in acoustic reproductive behaviors. When acoustic cues reach the antenna, sound-induced vibrations of the flagellum are transduced into electrophysiological signals by the Johnston's organ (JO), located in the second antennal segment within the pedicel. These signals are transmitted through the antennal nerve to the brain (Cator et al., 2009; Clements, 1963; Roth, 1948). Flagellar ears such as the antenna, however, have long been assumed to be restricted to sound detection in the acoustic ‘near field’, which occurs only in close-range interactions (Albert and Kozlov, 2016; Römer, 2020). Antennal hearing was therefore assumed not to be involved in detection of frog calls by frog-biting midges and mosquitoes, a process that involves hearing distant sound sources. It has been shown, however, that mosquito antennae can detect sound produced in the acoustic ‘far field’, when the sound source is located 10 m away (Menda et al., 2019). If the antenna can detect sound in the ‘far field’, it is to be expected that mosquitoes rely on antennal hearing for foraging. Although this rationale suggests that mosquitoes might use their antennae in contexts different from mating, such as predator avoidance or foraging, there is a gap in our knowledge about how mosquitoes may use hearing in non-mating contexts.

The antennae of mosquito species that rely on sound for mating exhibit common morphological and mechanical features across genera (Su et al., 2018). Males have plumose antennae mechanically tuned to detect and amplify the female's wingbeat frequency. Females, in contrast, have longer and less plumose antennae that are mechanically tuned to lower frequencies that do not match the male's wingbeat frequency. Unlike these species, mosquitoes that do not use hearing for mating do not exhibit sexual dimorphism in the general features of the antennal flagellum (e.g. length, plumosity). In mosquitoes, hearing conspecific mates has shaped antennal morphology modulating biomechanical and neurophysiological responses. However, it is unclear how the use of acoustic cues in other contexts may have shaped the characteristics of their antennae. Here, we examined how antennal ears are used beyond mating, concentrating on the use of mosquito hearing for foraging. We investigated mosquito hearing in a non-mating context by assessing the behavior, characterizing the flagellar morphology, and evaluating the neurophysiological and biomechanical response of the antennae of the frog-biting mosquito species *Uranotaenia lowii* (Fig. 1).

Although studies performed in the field show that *Ur. lowii* females hunt frogs by hearing their calls (Borkent and Belton, 2006; Lopez-Méndez et al., 2015; Reeves et al., 2018), the phonotactic behavior of this species has not been evaluated in controlled conditions. Similarly, there is no information about its mating behavior or the characteristics of its antennae. Owing to the limited information on different aspects of *Ur. lowii* biology, we first characterized their antennal morphology and investigated their phonotactic and mating behavior. Secondly, we examined the mechanical response of the antenna of *Ur. lowii* and contrasted it with the biomechanical response of species that use their antennae for hearing during mating. Finally, we took the first steps into investigating the neural response of the Johnston's organ of female *Ur. lowii* to identify their hearing frequency range to examine whether their antenna could detect frog calls. By integrating behavioral, biomechanical and neurophysiological approaches, we uncovered the unique acoustic behavior of *Ur. lowii* and investigated how antennal hearing is used to exploit the communication system of frogs.

## MATERIALS AND METHODS

We investigated the mating behavior, antennal vibration and neurophysiological response to sound of the frog-biting species *Uranotaenia lowii* Theobald 1901 using mosquitoes from our previous colony at Texas Tech University (Lubbock, TX, USA) and our current colony at Purdue University (West Lafayette, IN, USA). Both colonies were established from egg rafts obtained from the USDA-ARS-CMAVE Mosquito and Fly Research Unit (Gainesville, FL, USA). Larvae were fed using a 3:2 ratio of bovine liver powder and brewer's yeast. Adult mosquitos were reared in an insect cage (BioQuip lightweight aluminium collapsible cages; 46×46×46 cm; 2×2 mm mesh size) under controlled environmental conditions (22– 27°C, 50–80% relative humidity). Adults had constant access to a source of 10% (w/v) sucrose solution, and females were also blood-fed with cane toads (*Rhinella marina*) or Cuban treefrogs (*Osteopilus septentrionalis*) to support egg production.

### Antenna morphology characterization

External antenna morphology of six males and six females was characterized using optical microscopy (microscope: H550 S 10X Nikon; camera: Ds-Fi2, Nikon). We combined these results on the antenna of *Ur. lowii* with published studies on four mosquito species that use acoustic cues for mating (Göpfert and Robert, 2000; Simões et al., 2016, 2017; Staunton et al., 2020; Su et al., 2018), to create a comparative framework of morphological and mechanical features of the antenna across mosquito species.

### Mating behavior characterization

To examine mating swarm formation, security cameras (EV-2706-N3GQ, DVR: DR-104Q, Enforcer) installed at our *Ur. lowii* colony recorded the behavior of males and females 24 h a day, 7 days a week for 1 month. Also, by performing opportunistic visual surveys, we investigated the mating sequence of this species. To further characterize the flight trajectory of males and examine potential cues used in courtship, we performed behavioral observations in small experimental arenas (cylindrical plastic cup 8.3×14.3 cm high), containing a Petri dish (5.1 cm diameter) filled with water. Using custom-made MATLAB trajectory reconstruction algorithms, we analyzed the approach trajectory of 19 males prior to copulation attempts from video recordings. The directionality of the male approach was characterized using the orientation efficiency of trajectories calculated as the ratio between initial distance and path length, expressed as a percentage (Benhamou, 2004).

### Evaluation of female phonotaxis in response to frog calls

**Fig. 2.**
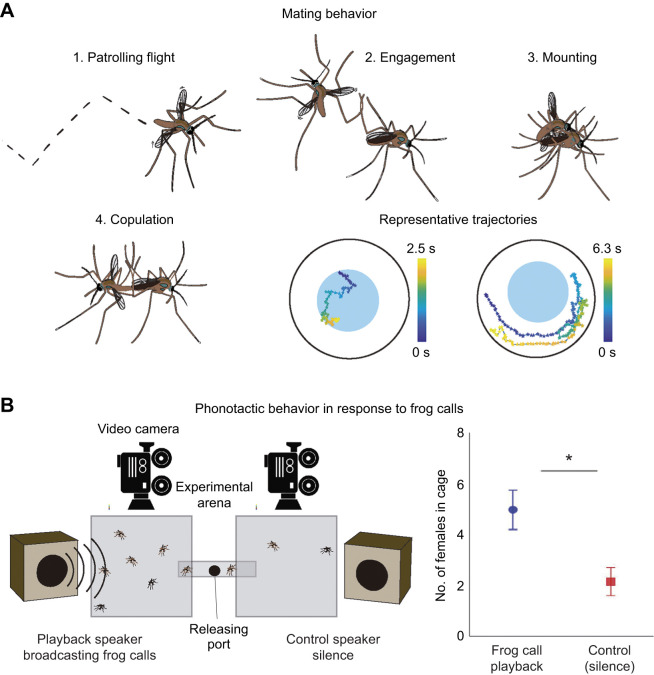
**Mating and phonotactic behavior of frog-biting mosquitoes *Uranotaenia lowii.*** (A) Stages and representative motion trajectories in the non-acoustic mating behavior of frog-biting mosquitoes *Uranotaenia lowii*. (1) Zigzag flying pattern and representative trajectories followed by males looking for females in a circular experimental arena (black outline) with a Petri dish filled with water (blue inside circle). Colour of the trajectory indicates the time when the male was moving as shown by the colour bars. (2) Initial contact between a male and a female. (3) Attempt of genital contact. (4) Copulation posture. Illustrations made by Ana M. Ospina-L. (B) Experimental setup and results of 18 phonotaxis trials in response to frog call playbacks. The right panel shows the number of females (mean±95% CI) moving to the cage with the frog call playback or a silent speaker (control) (paired *t*-test, *t*_17_=6.480, *P*<0.01).

Upon emergence, female *Ur. lowii* were separated into an empty insect rearing cage (30×30×30 cm^3^) and given a continuous supply of sucrose solution, which was removed 24 h before the experiment. Their phonotaxis behavior was studied inside a dark anechoic chamber (background noise level: 52 dB re. 20 μPa, 25°C and 52% relative humidity). Following Bartlett-Healy et al. (2008), the setup consisted of two insect cages (30×30×30 cm^3^) connected by a cylindrical tube (30 cm long×12 cm diameter) with a 2.5 cm hole in the center to introduce the mosquitoes. Two speakers (ScanSpeak Discovery 10F/4424G00, 89.5 mm diameter driven by a Pyle PTA1000 amplifier and a laptop) were each placed behind one cage (Fig. 2B). One speaker broadcast calls of the barking treefrog (*Dryophytes gratiosus*, formerly *Hyla gratiosa*) (90 dB re. 20 μP at 50 cm), while the other speaker was used as a control (plugged-in speaker with no call). Sound pressure level (SPL) was recorded using a Brüel & Kjær ½ inch microphone, Type 4188 attached to a sound level meter, Type 2238 (Brüel & Kjær, Naerum, Denmark). Each trial was recorded under infrared (IR) light using with one video camera (Sony Handycam, FDR-AX33) placed over each cage with additional IR lights. Ten females of 7–14 days old were tested at the same time during 18 trials for a total of 180 females. The trials lasted 15 min after releasing the females in the tube. At the end of the trial, the number of females in each cage and those that remained in the tube were counted. To avoid side biases, the location of the frog calls (left or right chamber) alternated between trials.

### Vibrational response of the antenna and flight-tone recording

**Fig. 3.**
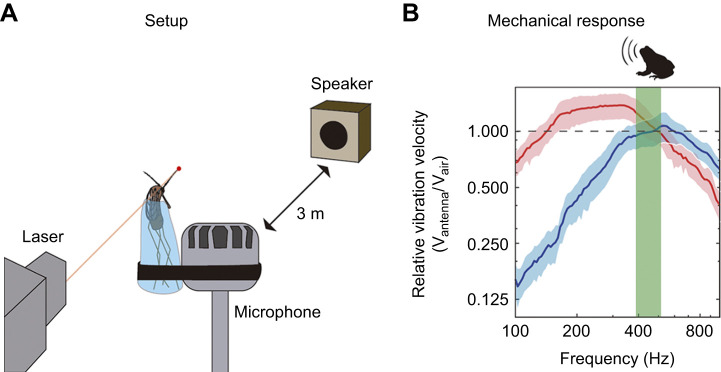
**The mechanical response of the female's antenna promotes detection of frog calls.** (A) Experimental setup inside an anechoic chamber. A laser Doppler vibrometer records the mechanical response of the antenna to acoustic stimuli broadcast by a speaker located 3 m from the specimen. The specimen is attached to a reference microphone to allow for comparisons between antennal and air velocities. (B) Antennal mechanical responses (means±s.d.) of six females (red) and seven males (blue) relative to the air particle velocity recorded by the microphone. Female antennae amplify (values above dotted line) the dominant frequency of the call of barking treefrogs (green shadow). The female's antenna is hence mechanically tuned to lower frequencies (antenna resonance frequency, *t*-test, *t*_6,7_=–16.87, *P*<0.01) and shows higher levels of amplification (amplification factor, *t*-test, *t*_6,7_=2.49, *P*=0.03). Axes are presented in log scale.

Laser Doppler vibrometry was used to assess the vibrational response of the antennae of males and females *Ur. lowii*. Recordings were performed inside an anechoic chamber at the Watson School of Engineering and Applied Sciences at Binghamton University (NY, USA). The chamber is anechoic at all frequencies above 80 Hz and has a noise floor of ∼0.5 dBA SPL. The main resonant frequency of the antennae was established by recording flagellum vibrations in response to tones of 90 dB re. 20 μPa SPL with frequencies across the range covered by mosquito flight tones (0.1–1 kHz). A single-dot laser Doppler vibrometer (OFV-534, Polytec Inc.) was used to record vibrations from the tip of the antennae of six females and seven males in response to acoustic stimuli broadcast by a loudspeaker (HR 824, Mackie) located 3 m from the specimen and adapted to obtain plane wavefronts (Zhou and Miles, 2017) (Fig. 3A). In addition, we used a calibrated ¼ inch microphone (4135, B&K) set by the mosquitoes to measure the sound pressure as experienced by the individual. Air particle velocity *u*(*t*) was calculated from *u*(*t*)=*p*(*t*)/ρ_0_*c* (Zhou and Miles, 2017), where *p*(*t*) is the measured sound pressure, ρ_0_ is the density of air and *c* is the speed of sound in air. We obtained the maximum resonance peak of the vibration velocity of the antenna tip, using air particle velocity as a reference.

We recorded the flight tones of 22 female and 17 male free-flying *Ur. lowii.* Recordings were performed by introducing one mosquito at a time in plastic vials (3.5 ml) containing an electret microphone (FG-23329-C05, Knowles), sensitive to intensities higher than −53 dB (relative to 1.0 V/0.1 Pa). In addition, following the recording protocol described in Mukundarajan et al. (2017), a cell phone was placed inside the rearing cage of the colony to examine the frequency distributions of the flight tones of male and female flying freely around their rearing cage. All recordings were performed at 25±1°C.

### Neural response of the antenna

**Fig. 4.**
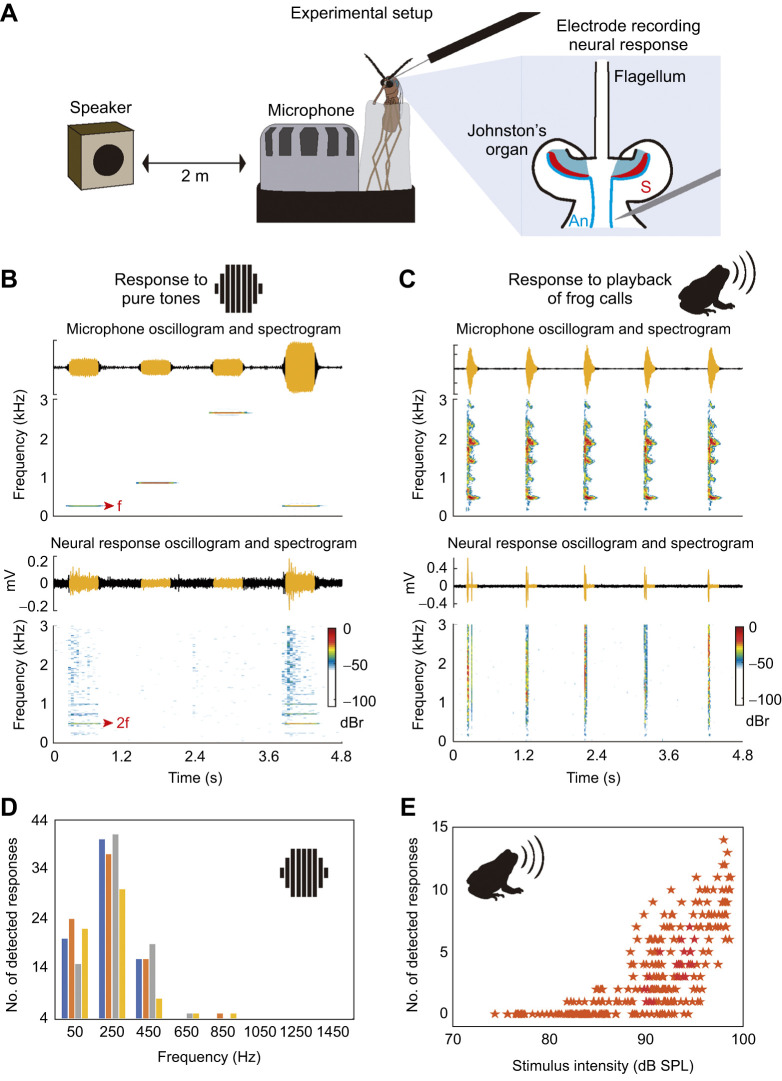
**Frog-biting mosquitoes detect frog calls with their antennae.** (A) Experimental setup. Sensory neurons (S) transform sound-induced vibrations into electrophysiological signals that travel through the antennal nerve (An). Neural responses were recorded by an electrode at the antennal nerve. (B,C) Oscillograms and spectrograms of representative recordings of acoustic stimuli and neural signals obtained in response to pure tones (B) and frog calls (C). Yellow segments in oscillograms indicate acoustic stimuli and their respective neural responses. The doubling frequency (2f, red arrow) of phasic responses is indicated in the spectrogram and spikes are shown in the oscillogram. (D) Number of responses detected in four females in response to pure tone stimuli varying frequency and intensity (results for intensity can be found in Fig. S2). Each color bar corresponds to an individual female mosquito. (E) Number of neural responses detected in response to playback of frog calls.

Following previous work (Menda et al., 2019), we investigated the response of the antennae of *Ur. lowii* females by recording extracellular compound action potentials at the antennal nerve. Mosquitoes were gently inserted into a pipette tip and fixed in place with Kerr dental sticky wax (58°C melting point). This approach restrains the mosquito, confining any movement of its head and proboscis, so the antennae are the only appendages that can move freely without compromising their physical condition. Neural responses were recorded by inserting tungsten microelectrodes (4 MΩ, MicroProbes, WE30014.0F5) through the thin layer of cuticle located below the pedicel (Fig. 4A). Signals were amplified (head stage and amplifier 1800, A-M Systems), digitized at 20 kHz and analyzed using MATLAB custom-made algorithms. Phasic and spiking responses were detected using thresholding schemes. Following Warren et al. (2009), the threshold to detect phasic responses was established as the SPL necessary to produce a receptor potential of 5 dB above the recording of non-stimulated JO. Spikes were detected when exceeding two scaled median absolute deviations from the median of the recording noise floor. We confirmed that responses were generated by the flagellum vibration by carefully adding a tiny drop of water that constrained the flagellum's motion and consequently blocked the neural responses. Once the drop of water dried out, the response was detected again.

Two playback experiments were performed. First, we estimated the frequency range that elicits neural responses at the antennal nerve. We assessed the JO response in a frequency range between 0.05 and 4 kHz by stimulating the antenna with pure tones that varied in frequency at 0.2 kHz intervals (trapezoid shaped, 300 ms duration, 50 ms rise and fall time; 200 ms between tones). Pure tones were played in pseudo-random order by a loudspeaker located at 3 m from the specimen, at a range of sound intensity levels between 65 and 90 dB SPL (re. 20 μPa, measured at the mosquito's position). Second, we tested whether calls of the barking treefrog are detected by the JO, as this acoustic stimulus prompts phonotaxis responses of female *Ur. lowii* in nature (Borkent and Belton, 2006) and in the lab (present study). To analyze the neural response elicited by these calls, we performed playback experiments varying the sound level of stimuli between 72 and 104 dB SPL. Neural activity was recorded at the antennal nerve of seven individuals. Background noise in the room had an average intensity of 61.55±2.47 dB SPL.

### Data analysis

All analyses were conducted using MATLAB (MathWorks). Results of variables normally distributed are presented as means±s.d. and *t*-tests were used to compare between groups. Variables that followed non-normal distributions are presented using medians and interquartile range (IQR). No comparisons were performed between variables following non-normal distributions. Data and codes that support the findings of this study are available from the Purdue University Research Repository (doi:10.1016/j.cub.2016.09.017).

## RESULTS

### Antenna morphology

The antenna of *Ur. lowii* exhibits evident differences in morphology compared with the antenna of mosquito species that use hearing for mating. In contrast to species that use hearing for mating, where the antennae of males are plumose and shorter than the antennae of females, in *Ur. lowii*, the flagellum is not sexually dimorphic (Fig. S2). In *Ur. lowii*, the antennal flagellum of males is not plumose, and its length is comparable to that in females (females: 1.47±0.06 mm, males: 1.43±0.03 mm; *t*-test: *t*_5,5_=1.35, *P*=0.21). Although sexual dimorphism is not evident in the flagellum of *Ur. lowii*, closer examinations of the pedicel, which contains the JO, revealed sexual differences. Specifically, in males, the insertion point where the flagellum is attached to the JO is deeper, and has an hourglass-like shape, compared with the shallow, flat structure of females (Fig. S1). This sex-specific shape of the attachment of the flagellum resembles the pedicel of species that use hearing for mating.

### Non-acoustic mating behavior

Although *Ur. lowii* females hunt frogs by hearing their calls (Borkent and Belton, 2006), we found no evidence that acoustic signals mediate the mating behavior in this species. Our visual surveys revealed the absence of aerial aggregations, a breeding strategy closely associated with the use of acoustic mating signals in flies (Downes, 1969). Viable eggs, however, were regularly laid regardless of the absence of aerial aggregations. Our observations confirmed that *Ur. lowii* mosquitoes mate without males forming swarms and instead, copulation occurs on the ground. Of the 69 mating events observed, none of them occurred in the air. Copulation occurred primarily on (40%) or near (60%) water sources, as soon as 30 min post-emergence. Mating behavior is initiated by a single male flying close to the ground following zig-zag patterns (Fig. 2A). This behavior is not synchronized among individuals and can occur at any time of day or night (with or without light). Before mating attempts were initiated, males and females were inactive for long periods of time (over 5 min). No distinctive behaviors of females were associated with the initiation of a male's approach. Patrolling time varied highly between males (range: 1.0–6.3 s, median: 2.5, IQR: 1.4–4.3) and males did not follow a direct path to the female (orientation efficiency median: 33%, IQR 20–45%). Once a female is reached, the male intertwines one of his legs with one of her legs (Fig. 2A) and struggles to position himself behind her. In this position, the male attempts genital contact from behind by lowering his abdomen (Fig. 2A). However, in most mating attempts (1058 out of 1127), females rejected males by kicking them away. Rejected males attempted to mate again with the same female in about 10% of the cases (106 out of 1058 rejections), but more often they flew away to initiate contact with another female (952 out of 1058 rejections). If genital contact is successful, the male twists his body, while maintaining genital contact, until the couple reaches an end-to-end mating position (Fig. 2A). During copulation, males hold their wings extended at a 90 deg angle away from their body. Females, in contrast, hold their wings next to their body, in a resting position. In all the cases in which males were able to invert themselves, copulation continued and lasted for over 1 h (66.8±22.3 min, *n*=69). Copulation finished abruptly by males suddenly detaching their genitalia and moving away without apparent behaviors performed before copulation ended.

### Female phonotaxis in response to frog calls

Female *Ur. lowii* exhibit positive phonotaxis responding to barking treefrog calls in laboratory conditions. This result supports studies in the wild of females from this mosquito species using audition to locate their anuran host (Borkent and Belton, 2006; Lopez-Méndez et al., 2015). We found a significant effect of frog call playback on mosquito motion. Overall, 50.0% of the females moved towards the playback of frog calls, whereas 21.7% of the females moved towards the silent cage (paired *t*-test, *t*_17_=6.480, *P*<0.01; Fig. 2B). No effect of the position of cages (left or right) was found (*t*-test, *t*_16_=1.940, *P*=0.07). Phonotaxis was not evaluated for males as they do not feed on frogs and have not been captured using acoustic traps in the field.

### Mechanical response of the antenna of male and female frog-biting mosquitoes

The mechanical response of the antenna of *Ur. lowii* differs from species that use hearing during mating. In those species, three characteristics are associated with the detection of flight tones: (1) males broadcast flight tones with higher frequencies than females, (2) the antenna of females resonates at lower frequencies than the antenna of males, and (3) the antenna of males resonates at frequencies close to the fundamental frequency of the female's flight tone. Only two of these characteristics are present in *Ur. lowii*. First, like species that rely on sound for mating, males broadcast flight tones of higher frequencies than females (females: 824.9±66.8 Hz and males: 1070.5±66.8 Hz; *t*-test, *t*_16,21_=11.1, *P*<0.01). Second, the mechanical response of the antenna differs between sexes (Fig. 3B)*.* The female's antenna amplifies, on average, frequencies between 141.2 and 506.9 Hz, with a maximum amplification factor of 1.38±0.20 at its resonant frequency (331.5±15.6 Hz). The male's antenna amplifies higher frequencies, in a range between 468.0 and 594.7 Hz, with a maximum amplification factor of 1.08±0.17 at its resonance frequency (543.36±22.04 Hz). The female's antenna is hence tuned to lower frequencies (antenna resonance frequency, *t*-test, *t*_6,7_=–16.87, *P*<0.01) and shows higher levels of amplification (amplification factor, *t*-test, *t*_6,7_=2.49, *P*=0.03). Although these characteristics are similar to species that use hearing during mating such as *Aedes aegypti*, *Anopheles gambiae*, *Culex quinquefasciatus* and *Toxorynchites brevipalpis* (Staunton et al., 2020; Su et al., 2018; Simões et al., 2017, 2016 and Göpfert and Robert 2000), the antennal resonance of males in *Ur. lowii* is at a frequency distant from the fundamental frequency of the female's flight tone. The female's antenna, however, amplifies a range of frequencies that covers the peak frequency of calls of the barking treefrog (Fig. 3B).

### Detection of frog calls by the antenna of female frog-biting mosquitoes

Phasic and spiking responses were detected at the antennal nerve of four females in response to tones. Phasic responses exhibited a dominant frequency twice as high as the stimulus frequency (Fig. 4B). This frequency doubling is a common feature of extracellular recordings performed at the antennal nerve of mosquitoes (Windmill et al., 2018). An evaluation of the SPL sufficient to elicit compound potentials 5 dB above the recording noise floor shows that female *Ur. lowii* are sensitive to frequencies up to 450 Hz, with minimal responses at higher frequencies (Fig. 4D). The minimum intensity threshold to provoke detectable responses was 68.9±0.7 dB SPL (∼10 dB SPL over the noise floor) and occurred at 250 Hz. Parameters associated with response intensity such as the magnitude of the doubling frequency (2f) phasic component varied in proportion to the sound level of acoustic stimulation (Fig. S2).

In our second experiment, we detected robust neural responses elicited by calls of the barking treefrog (Fig. 4C). Calls consisted of harmonic series with a fundamental frequency that decreased from ∼470 to ∼410 Hz (Fig. 4C) and had an average duration of 0.15±0.01 s. Playbacks with intensities higher than 81.8 dB SPL elicited spiking responses and the number of spikes increased with the sound level of the stimuli (Fig. 4E).

## DISCUSSION

Our results show that *Ur. lowii* has a unique acoustic behavior among mosquitoes and midges. That is, in this species, females rely on acoustic cues for host-seeking behavior, but neither sex uses conspecific flight tones during courtship behavior. This unique acoustic behavior of *Ur. lowii* provides an ideal opportunity to investigate mechanisms underlying hearing in the context of exploitation of frog calls, without confounding factors associated with the use of sound for mating.

It is unknown whether the acoustic and mating behavior of *Ur. lowii* is found in other species from the Uranotaeniini tribe, but it does not seem characteristic of this clade. Several *Uranotaenia* species form swarms and mate in the air (Corbet, 1964; Corbet and Haddow, 2009), behaviors associated with the use of sound during courtship. The reproductive behaviors and morphological characteristics of the antenna of *Ur. lowii* that we report here are uncommon in the Uranotaeniini tribe and resemble instead those of a distantly related mosquito species, *Culiseta inornata* (Culisetini). In this species, like in *Ur. lowii*, the antennae are not sexually dimorphic, and males use contact pheromones rather than acoustic cues to find females (Kliewer et al., 1966; Lang, 1977). We propose that a similar strategy, based on chemical communication, may be used by male *Ur. lowii* to find females. Visual cues, however, could also play a role in their mating sequence. Further investigation of the mechanisms that meditate courtship behavior in *Ur. lowii* is necessary to broaden our understanding of the biology of this species.

In line with the evidence from species that do not use acoustic strategies for mating (Downes, 1969; Keith Philips et al., 1996; Lang, 1977; Zsemlye et al., 2005), the antennae of *Ur. lowii* males are not plumose. Our results show, however, that acoustic traits such as those associated with the detection of flight tones for mating are present in *Ur. lowii*. First, males produce flight tones with higher frequency compared with females. Second, the male's antenna is mechanically tuned to higher frequencies than the female's antenna. Together, these results suggest that sexual differences in the morphology of the pedicel and the resonance of the antenna are traits that persist in *Ur. lowii* despite the evolutionary loss of acoustically mediated mating. The mechanical response of the male's antenna, however, differs from species that use hearing for mating as its resonance does not match the flight-tone frequency of females. Similarly, the antenna of *Ur. lowii* males shows, at its resonance frequency, lower amplification levels (1.07±0.20 measured at the tip of the antenna) compared with mosquito species using acoustic mating signals, such as *Aedes aegypti* (3.0±0.3) (Göpfert et al., 1999) or *Toxorhynchites brevipalpis* (4.3±0.4) (Göpfert and Robert, 2000). The lower hearing sensitivity of *Ur. lowii* males is likely due to their less plumose antennae, as antennal hairs influence acoustic sensitivity (Göpfert et al., 1999). Both the mismatch between male hearing and female signals as well as the lower sensitivity of male antennae are consistent with the loss of acoustic mating behaviors in this species.

Females of *Ur. lowii* exhibit phonotaxis to frog calls. This result supports previous findings obtained under field conditions, where females were captured using acoustic traps (Borkent and Belton, 2006; Lopez-Méndez et al., 2015; Reeves et al., 2018). In this study, however, we showed that *Ur. lowii* from Florida, where barking treefrogs also occur, are attracted to their calls, providing an ecologically valid perspective of their interaction. In addition, we examined phonotaxis under control conditions excluding potential alternative explanations to conclusively demonstrate that frog calls are sufficient to elicit attraction. Although other sensory cues may modulate short-range host selection, our findings, combined with previous field studies (Borkent and Belton, 2006; Lopez-Méndez et al., 2015), suggest that frog calls provide the main long-distance cue used by *Ur. lowii* to locate their anuran host.

**Fig. 5.**
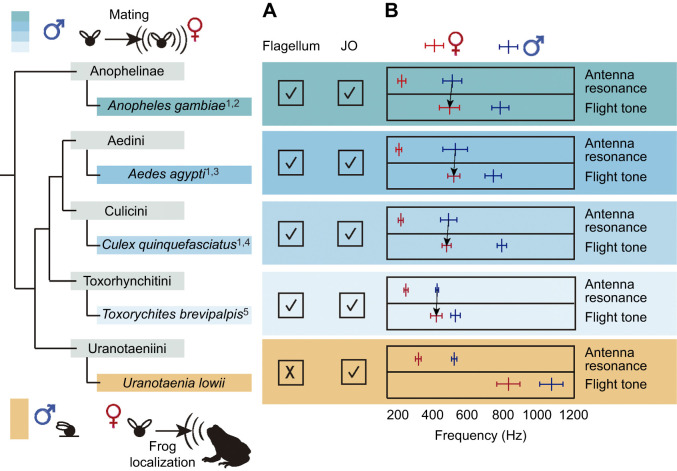
**The mechanical response of frog-biting mosquito antennae shares similar traits to non-frog-biting species despite differences in morphology and flight tones.** Morphological and mechanical characteristics of the antennae of frog-biting mosquitoes (orange) and species that use flight tones for mating (blue). Data adapted from: (1) Su et al. (2018), (2) Simões et al. (2017), (3) Staunton et al. (2020), (4) Simões et al. (2016) and (5) Göpfert and Robert (2000) (references are indicated as superscripts to species names). (A) Presence (✓) or absence (X) of sexual dimorphism in the flagellum and Johnston's organ (JO) insertion point are shown. Microscopy of the antenna is included in Fig. S2. (B) Flight-tone frequency and antenna resonance (means±s.d.) indicated for females (red) and males (blue). Arrows connect the antenna and acoustic signal for which there is evidence of phonotaxis in mating.

Although the bioacoustic behavior of *Ur. lowii* is uncommon, the mechanical response of the antennae in males and females resembles that of species using sound for mating, albeit with certain distinctive features. The antenna of females *Ur. lowii* resonates at lower frequencies than the male's antenna, following the pattern of species that use flight tones for mating (Fig. 5). In this species, however, the range of frequencies that the antenna detects also matches the dominant frequency of the barking treefrog call. Our results support the prediction that the mechanical response of the female's antenna allows for the amplification and detection of frog calls (Borkent and Belton, 2006). Together, these results from both males and females suggest that despite the lack of use of sound in mating, features that promote hearing to mate in the air using flight tones still prevail in the antenna of *Ur. lowii*, and drive the detection of frog calls.

Encoding the vibration of the flagellum by the neural system is the next step of sound detection. Acoustic stimuli elicited phasic and spiking responses at the antennal nerve of female *Ur. lowii*. Phasic responses transmit an analog representation of the acoustic stimulus containing frequency and phase information. Our results show that both responses are elicited by low-frequency tones and that there is a positive relationship between SPL of acoustic stimulation and the magnitude of neural responses. We detected clear responses at low frequencies up to 450 Hz. Although the resolution of our experiment was not high enough to determine the exact limits of audition in this species, the results presented here show that the JO of this frog-biting species responds to low frequencies and the characteristics of the neural signal are similar to those of species that use sound for mating (Lapshin, 2013). Playbacks broadcasting frog calls with SPLs higher than 85.3 dB also elicited responses at the antennal nerve. This SPL is equivalent to those produced by a barking treefrog males calling at a distance of 2 m (Feng et al., 1976). Our results thus provide, for the first time, neurophysiological evidence supporting the hypothesis that the antennae of female frog-biting mosquitoes are the sensory sensors used for detecting frog calls.

The similarity in the vibrational and neural response of the antenna between frog-biting and non-frog-biting mosquitoes suggests there may be other relevant commonalities between these sensory systems. For instance, frequency intermodulation, which is a critical trait that allows male mosquitoes to detect females (Ingham et al., 2018; Simões et al., 2016; Warren et al., 2009), may also be relevant for detecting frog calls. When females forage, exploiting the communication system of frogs, intermodulation between the sound produced by their own wingbeat and the frequency components of frog calls might generate distortion products. This effect likely improves frog call detection by reducing the intensity threshold and increasing sensitivity across frequencies (Lapshin and Vorontsov, 2013). Further studies that investigate the role of flying when hearing frog calls are necessary to examine the hypothesis that intermodulation also mediates how frog-biting mosquitoes hear frog calls.

Together, our results in the context of the use of sound within Culicidae, and more broadly within the infraorder Culicomorpha, are in line with the hypothesis proposing that the ability of frog-biting mosquitoes to eavesdrop on anurans was evolutionarily co-opted from using acoustic signals for mating (de-Silva et al., 2015). By examining antennal morphology, biomechanics and neurophysiology in an eavesdropping mosquito that limits audition to hearing frog calls and contrasting our findings with those from non-eavesdropping counterparts (Fig. 5), this study provides the first comparative evidence for this ‘antennal co-option hypothesis’. Although sensory biases in receivers have been widely investigated (Christy et al., 2003; Nakano et al., 2015; Proctor, 1992; Rodd et al., 2002; Ryan and Rand, 1990; Ter Hofstede et al., 2015), the present study presents a scenario in which selection for mating promotes the exploitation of a novel resource. Ultimately, this study highlights how pre-existing features of sensory systems that evolved for mating could promote the exploitation of other resources, a phenomenon likely to be widespread across organisms.

### Conclusions

We provided compelling morphological and biomechanical evidence indicating that *Ur. lowii* females can use their antennal ears to eavesdrop on frog calls. Unlike most mosquito species, however, *Ur. lowii* do not use acoustic mating signals. Despite the loss of acoustic mating signals, our results suggest that ancestral traits associated with hearing conspecific flight tones in the context of mating prevail in their antennae and, in the case of females, allow the detection of frog calls. Here, we showed, for the first time, that antennae of female frog-biting mosquitoes are sensors capable of detecting distant frog calls by resonating at frequencies that promote the detection of these acoustic cues. Thus, this study revealed a novel scenario in mosquito bioacoustics, in which antennal hearing is used for purposes other than mating.

## Supplementary Material

10.1242/jexbio.245359_sup1 Supplementary information
