# Supplementary material for: Tiny spies: mosquito antennae are sensitive sensors for eavesdropping on frog calls
Source: J Exp Biol. 2023 Dec 11;226(24):jeb245359. doi: 10.1242/jeb.245359 (PMC10753488; doi:10.1242/jeb.245359)
Supplement: Supplementary information [file jexbio-226-245359-s1.pdf]

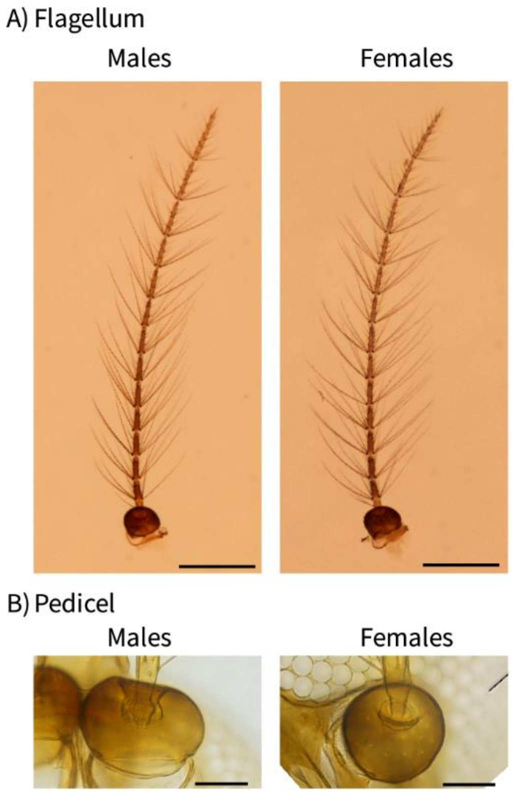

Fig. S1. Morphology of the antennal hearing organ of *Uranotaenia lowii*. Antenna flagellum (Scalebar 250 um) (A) and insertion point of the flagellum at the pedicel (B) between males and females (Scalebar 50 um). While there are no evident sexual differences in length of the flagellum or number of fine hairs, the insertion point where the flagellum is connected to the JO is sex specific. Males exhibit a deep, hourglass-shaped insertion point while females have a superficial, flat one.

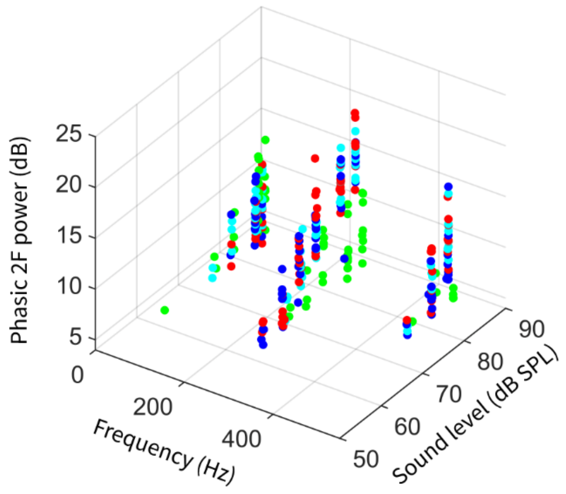

Fig. S2. Relationship between frequency, sound level of acoustic stimuli and magnitude of phasic responses. Colors indicate measurements obtained from four different individuals. The magnitude of phasic responses increases in proportion to sound level of stimulation.

Journal of Experimental Biology • Supplementary information

Table S1. References used to create Figure 1. Evidence of the use of sound across different contexts in mosquitoes (Diptera: Culicidae) and their relatives (Diptera Corethrellidae and Diptera: Chaoboridae).

Evidence of mating in-flight or the use of hearing for mating	Evidence of the use of hearing to localize frog calls
**Diptera: Corethrellidae**	
-Caldart VM, Beux M, Santos D, Lop S, Pinho C, Cechin SZ. (2016) Hematophagous flies attracted to frog calls in a preserved seasonal forest of the austral neotropics, with a description of a new Species of Corethrella (Diptera: Corethrellidae). Zoolog Sci, 527-536. (doi:10.2108/zs150173)	-McKeever S. (1977) Observations of Corethrella feeding on tree frogs (Hyla). Mosq. News 37, 522-523.
-de-Silva P, Bernal XE. (2013) First report of the mating behavior of a species of frog-biting midge (Diptera: Corethrellidae). Florida Entomol. 96, 1522–1529. (doi:10.1653/024.096.0434)	-Bernal XE, Rand AS, Ryan MJ. (2006) Acoustic preferences and localization performance of blood-sucking flies (Corethrella Coquillett) to túngara frog calls. Behav. Ecol. 17, 709–715. (doi:10.1093/beheco/arl003)
-de-Silva P, Nutter B, Bernal XE. (2015) Use of acoustic signals in mating in an eavesdropping frog-biting midge. Anim. Behav. 103, 45–51. (doi:10.1016/j.anbehav.2015.02.002)	-Bernal XE, De-Silva P. (2015) Cues used in host-seeking behavior by frog-biting midges (Corethrella spp. Coquillet). J. Vector Ecol. 40, 122–128. (doi:10.1111/jvec.12140)
**Diptera: Chaoboridae**	
-Moore MV. (1986). Method for culturing the phantom midge Chaoborus (Diptera: Chaoboridae) in the laboratory. Aquaculture 56, 307-316	-Toma T, Miyagi I, Higa Y, Okazawa T, Sasaki H. (2005) Culicid and Chaoborid flies (Diptera: Culicidae and Chaoboridae) attracted to a CDC miniature frog call trap at Iriomote Island, the Ryukyu Archipelago, Japan. Med. Entomol. Zool. 56, 65–71.
- Nielsen ET, Greve H. (1950). Studies on the swarming habits of mosquitos and other Nematocera. Bulletin of Entomological Research 41, 227-258.	
- Parma S. (1971). *Chaoborus flavicans* (Meigen)(Diptera, Chaoboridae), an autecological study. University of Groningen, Groningen, 1-128	
**Diptera: Culicidae**	
**Anophelinae**	
- Charlwood JD, Jones MDR. (1980). Mating in the mosquito, *Anopheles gambiae* sl. Physiological Entomology 5,315-320.	No data
-Simões PMV, Gibson G, Russell IJ. (2017) Pre-copula acoustic behaviour of males in the malarial mosquitoes *Anopheles coluzzii* and *Anopheles gambiae* s.s. does not contribute to reproductive isolation. J. Exp. Biol. 220, 379–385. (doi:10.1242/jeb.149757)	
-Gibson G, Warren B, Russell IJ. (2010) Humming in	

Journal of Experimental Biology • Supplementary information

tune: sex and species recognition by mosquitoes on the wing. J. Assoc. Res. Otolaryngol. 11, 527–40. (doi:10.1007/s10162-010-0243-2)	
-Quraishi MS. (1965). Swarming, mating, and density in nature of *Anopheles stephensi* mysorensis. Journal Econ. Entomol 58, 821-4	
- Van Somersen GC. (1976). A further note on swarming of male mosquitoes and other Nematocera in Kenya. Entomologist's Monthly Magazine. 111:147-60.	
-Pantoja-Sánchez H, Gomez S, Velez V, Avila FW, Alfonso-Parra C. (2019) Precopulatory acoustic interactions of the New World malaria vector *Anopheles albimanus* (Diptera: Culicidae). Parasit. Vectors 12, 386. (doi:10.1186/s13071-019-3648-8)	
**Ficalbiini**	
-Corbet PS. (1964). Observations on the swarming and mating of mosquitoes in Uganda. Proc. R. Entomol. Soc. A, General Entomology, 39 (1–3), 15–22. (https://doi.org/10.1111/j.1365-3032.1964.tb00777.x)	No data
**Hodgesiini**	
No data	No data
**Orthopodomyiini**	
No data	No data
**Toxorhynchitini**	
-Gibson G, Russell I. (2006) Flying in tune: sexual recognition in mosquitoes. Curr. Biol. 16, 1311–6. (doi:10.1016/j.cub.2006.05.053)	No data
**Culisetini**	
-Pletzen R, Van Der Linde T. (1981). Studies on the biology of *Culiseta longiareolata* (Macquart) (Diptera: Culicidae). Bull. Entomol. Res., 71(1), 71-79. doi:10.1017/S000748530005104X	No data
- Lindegren JE. (1966) Culture techniques for permanent colonization of *Culiseta incidens* (Thomson) (Diptera:Culicidae). Mosquito News (26) 3, 63-65	
**Aedini**	
- Lounibos LP. (1980). The bionomics of three sympatric Eretmapodites (Diptera: Culicidae) at the Kenya coast. Bulletin of Entomological Research 70,309-320	No data
- Rees DM, Lawyer PG, Winget RN. (1971) Colonization of *Leptoconops kerteszi* Kieffer by anautogenous and autogenous reproduction (Diptera: Ceratopogonidae).	

Journal of Experimental Biology • Supplementary information

Journal of Medical Entomology 8(3), 266-271.	
-Cator LJ, Arthur BJ, Harrington LC, Hoy RR. (2009) Harmonic convergence in the love songs of the dengue vector mosquito. *Science* **323**, 1077–9. (doi:10.1126/science.1166541)	
- Bullini, L. & Coluzzi, M. (1980). Ethological mechanisms of reproductive isolation in *Culex pipiens* and *Aedes mariae* complexes (Diptera Culicidae). Monitore Zoologico Italiano 14, 99-100.	
- Nielsen, H.T. (1965). Swarming and some other habits of *Mansonia perturbans and Psorophora ferox* (Diptera: Culicidae). Behavioural Ecology: an Evolutionary Approach 42, 67-89.	
**Mansoniini**	
-Corbet PS. (1964) Observations on the swarming and mating of mosquitoes in Uganda. Proceedings of the Royal Entomological Society of London. Series A, General Entomology, 39(1–3), 15–22. https://doi.org/10.1111/j.1365-3032.1964.tb00777.x	
- Nielsen, H.T. (1965). Swarming and some other habits of *Mansonia perturbans and Psorophora ferox* (Diptera: Culicidae). Behavioural Ecology: an Evolutionary Approach 42, 67-89.	
**Culicini**	
- Ikesshoji T, Yap HH. (1987). Monitoring and chemosterilization of a mosquito population, *Culex quinquefasciatus* (Diptera: Culicidae) by sound traps. Applied Entomology and Zoology 22, 474-481	- Bartlett-Healy K, Crans W, Gaugler R. (2008) Phonotaxis to Amphibian Vocalizations in *Culex territans* (Diptera: Culicidae). Ann. Entomol. Soc. Am. 101, 95–103. (doi:10.1603/0013-8746(2008)101[95:ptavic]2.0.co;2)
- Warren B, Gibson G, Russell IJ. (2009) Sex Recognition through midflight mating duets in *Culex* mosquitoes is mediated by acoustic distortion. Curr. Biol. 19, 485–91. (doi:10.1016/j.cub.2009.01.059)	
-Simões PMV, Ingham RA, Gibson G, Russell IJ. (2016) A role for acoustic distortion in novel rapid frequency modulation behaviour in free-flying male mosquitoes. J. Exp. Biol. 219, 2039–2047. (doi:10.1242/jeb.135293)	
**Sabethini**	
-Armbruster, P., Bradshaw, W. E., Steiner, A. L., & Holzapfel, C. M. (1999). Evolutionary responses to environmental stress by the pitcher-plant mosquito, *Wyeomyia smithii*. Heredity, 83(5), 509–519. https://doi.org/10.1038/sj.hdy.6886040	No data
**Aedeomyiini**	
No data	No data
**Uranotaeiini**	

Journal of Experimental Biology • Supplementary information

-Haddow AJ, Corbet PS. (1961) Entomological studies from a high tower in Mpanga Forest, Uganda. Transactions of the Royal Entomological Society of London 113(11), 284-300.	-Camp J V., Bakonyi T, Soltész Z, Zechmeister T, Nowotny N. (2018) Uranotaenia unguiculata Edwards, 1913 are attracted to sound, feed on amphibians, and are infected with multiple viruses. Parasites and Vectors 11, 456. (doi:10.1186/s13071-018-3030-2)
-Corbet PS. (1964) Observations on the swarming and mating of mosquitoes in Uganda. Proc. R. Entomol. Soc. London. Ser. A, Gen. Entomol. 39, 15–22. (doi:10.1111/j.1365-3032.1964.tb00777.x)	- Lopez-Méndez R, Castillo-Martínez A, Covarrubias-Castro JL, Quetzaly K, Monserrat Cueto-Medina S, Ortega-Morales AI. (2015) Atracción de Uranotaenia lowii (Diptera: Culicidae) al sonido de cantos de ranas (Amphibia: Anura), en Tabasco, Mexico. Entomol. Mex. 2, 547–551.
-Corbet PS, Haddow AJ. (2009) Diptera swarming high above the forest canopy in Uganda, with special reference to tabanidae. Trans. R. Entomol. Soc. London 114, 267–284. (doi:10.1111/j.1365-2311.1962.tb01080.x)	- de Silva WAPP, Bernal XE, Chathuranga WGD, Herath BP, Ekanayake C, Abeysundara HTK, Karunaratne SHPP. (2020) Feeding patterns revealed host partitioning in a community of frog-biting mosquitoes. Ecol. Entomol. 45, 988–996. (doi:10.1111/een.12874)
	- Borkent A, Belton P. (2006) Attraction of female Uranotaenia lowii (Diptera: Culicidae) to frog calls in Costa Rica. Can. Entomol. 138, 91–94. (doi:10.4039/N04-113)

Journal of Experimental Biology • Supplementary information
